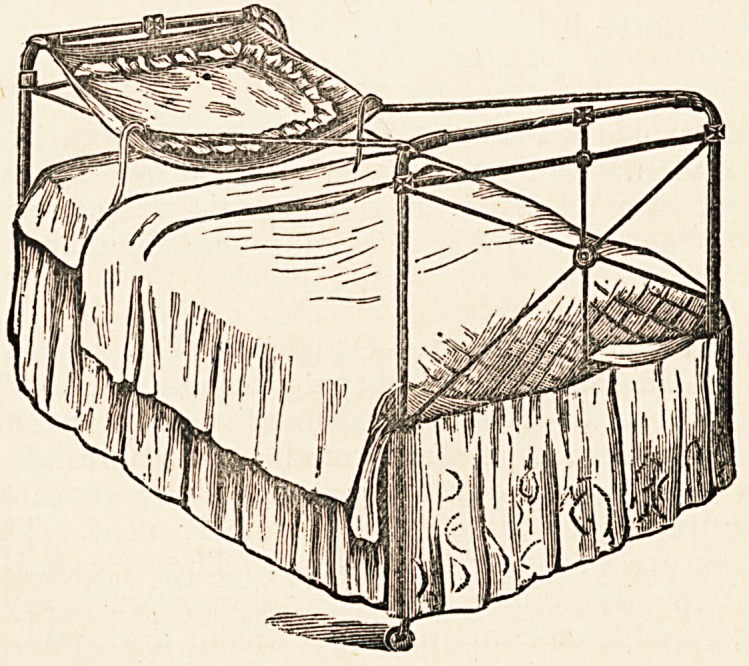# Notes on Preparations for the Sick

**Published:** 1896-06

**Authors:** 


### Notes on Preparations for the Sick.

**Lysidine.**—JEYES' SANITARY COMPOUNDS CO., LTD., London.—This new solvent for uric acid is the 50 per cent. aqueous solution of a crystalline but very hygroscopic organic base allied to piperazine, but having five times its solvent power on uric acid. Professor Gerhardt of Berlin speaks well of it. The dose is 30

grains, daily increasing to 150 grains. It is said to be best given in a pint of aerated water.

---

**Ethyl Phosphinate of Pyridin.**—Lorimer and Co., London.—This preparation is said by its promoters to possess "all the beneficial properties of **Pyridin,** whilst it is presented in an attractive and agreeable form, so combined that, whilst it destroys the bacilli, absorbs tubercle and allays irritation and inflammation, it also subdues cough and soothes the nervous system, whilst it checks night perspirations, improves the appetite, adds weight, and produces healthy lung-tissue; in short, it does in and by itself all that is wished for in the alleviation, arrest, and cure of consumption." No one who knows anything about phthisis will believe all this. Such statements are easy to make, but they are not easy of proof. We venture to maintain that few of the above theoretical statements have yet been proved, but we accept the statement that the fluid is attractive and agreeable.

---

**Vichy; "Big Rapids" Medicinal Water.**—Messrs. Ingram and Royle have sent us specimens of these. The old State springs of **Vichy,** and the ordinary brands emanating therefrom, need no description. The modern preparation, in tabloid form, the "Comprimés de Vichy," will be found a very convenient way of producing a more or less effervescing and warm glass of Vichy (*sources de l'état*) similar to the water as it comes from the natural spring. Three *comprimés* should be dissolved in a glass of ordinary warm water, or distilled water will give a more accurate imitation of the original. There appears to be no necessity for the importation of large quantities of plain or distilled water.

The "**Big Rapids**" sample is described as "the strongest natural mineral water known," and the bottle is marked "Clark's red electric natural medicinal water." The water should be potent. It is not a purgative, and it contains only traces of iron; but the chlorides of sodium, magnesium, and calcium are so abundant that the specific gravity, after diluting to 10 volumes, is as high as 1024. It is odourless, colourless, and clear, and it should be of service in all gouty conditions by favouring elimination. How a concentrated brine can be spoken of as "pleasant to the taste" we fail to understand. Sea water is infinitely more palatable.

---

**"Frame Food" Stamina Tablets.**—Frame Food Co., Ltd., London.—These tablets, flavoured with liquorice, are made of Frame Food extract, which contains 10 per cent. of soluble wheat phosphates. They are sustaining, and are easily carried in the pocket; hence their utility and convenience for travellers whose opportunities for ordinary meals may be inadequate. There is no danger that they will take the place of more ordinary foods at leisure moments, for they require efficient dental crushing machinery, without which the "meal in the waistcoat pocket" cannot prevent starvation in the midst of plenty.

---

**Beef Juice.**—JOHN WYETH AND BROTHERS, London.—This dark-red and somewhat viscid fluid has, after dilution to six times its bulk, a specific gravity of over 1040. It contains a high percentage of albumen, amounting, as estimated in the picric acid tube, to about 3½. Its bright arterial-blood colour, lost by boiling, and the appearance after much dilution of the spectroscopic bands, show the presence of hæmoglobin. Chemical analysis indicates the presence of kreatinin, glycogen, tyrosine, and salts (especially sodium chloride). This meat extract is clearly something more than a stimulant; it must possess a considerable nutrient value, and be of service when taste and sight do not raise any difficulties in its administration.

---

**Viking Meat Juice.**—The VIKING FOOD AND ESSENCE CO., London.—This preparation is a thick fluid having a sp. gr. of about 1240, and a red hæmoglobin colour. A teaspoonful in a wineglass of cold water makes a very palatable drink; but boiling water precipitates the soluble albumen, which is shown by the picric acid tube to amount to 1 per cent. Like all juices of this kind, the amount of nutrient available for tissue formation in a teaspoonful dose must be very small; but as a stimulant for the production of physiological energy, both experience and theory prove it to be useful.

---

**Bi-palatinoids of Fehling's Test.**—OPPENHEIMER, SON AND CO., LTD., London.—We have given these a good trial, but we cannot recommend them, as the organic pellicle causes some decomposition of the fluid in the absence of sugar.

---

**Cachets; Sinapine Tissue.**—COOPER AND CO., London.—These **Cachets**, of different sizes, of varying colours, and with coloured inscriptions, made with the "Finot" cachet machine "with cups," are the most elegant mode of mounting medicinal powders which we have hitherto seen. The tints of the cachets with inscriptions to match are extremely neat. The cachets themselves are of small dimensions. They are flexible, tasteless, and readily soluble, and their dimensions vary from the smallest, capable of containing 5 grains of antipyrin, up to the largest, containing 28 grains. Many drugs dissolve more easily and therefore act more quickly when taken in powder form in cachet rather than in compressed tabloid or coated pill.

**Sinapine Tissue** is an improved mustard plaster that never blisters the skin. It consists of a tissue-paper saturated with mustard, and has the advantages of being convenient, always ready, and not too powerful. It is supplied in sheets, from which a piece of the desired size can be cut.

---

**Plasters: Seabury's Rubber Adhesive; Mead's Rubber Adhesive; Belladonna; Cantharidal; Capsicum; Mustard; Muslin and Silk; Isinglass; Surgeon's Transparent Dressing.**—SEABURY AND JOHNSON, New York.—Specimens of these have been sent to us by Messrs. Fassett and Johnson, who are the agents in this country for them. They are all of excellent quality, and are conveniently sent out either on reels or enclosed in paper envelopes. They are known as Mead's or Seabury's plasters, and they are so prepared as to keep good in any climate. The **Belladonna Plasters** are perforated, and can be worn without the slightest inconvenience, the material on which they are spread being light and fitting closely to the inequalities of the skin. Plasters when prepared like these are indeed a revolution from the old-fashioned plasters formerly in use in this country, and which were usually spread by the surgeon himself, or his apprentice, as they were required.

---

**The Oriole Pillow Sling.**—LIPSETT AND CO., Liverpool.—This device, suggested by Miss Weatherly of Portishead, from her own experience of the things which add to the comfort of an invalid, is a simple but nevertheless a very decided improvement on the ordinary bed-rest. Its nature and the mode of its application can be seen from the illustration.

## Figures and Tables

**Figure f1:**